# Inulin Improves Postprandial Hypertriglyceridemia by Modulating Gene Expression in the Small Intestine

**DOI:** 10.3390/nu10050532

**Published:** 2018-04-25

**Authors:** Sophie Hiel, Audrey M. Neyrinck, Julie Rodriguez, Barbara D. Pachikian, Caroline Bouzin, Jean-Paul Thissen, Patrice D. Cani, Laure B. Bindels, Nathalie M. Delzenne

**Affiliations:** 1 Metabolism and Nutrition Research Group, Louvain Drug Research Institute, Université catholique de Louvain, B-1200 Brussels, Belgium; sophie.hiel@uclouvain.be (S.H.); audrey.neyrinck@uclouvain.be (A.M.N.); j.rodriguez@uclouvain.be (J.R.); barbara.pachikian@uclouvain.be (B.D.P.); patrice.cani@uclouvain.be (P.D.C.); laure.bindels@uclouvain.be (L.B.B.); 2 IREC Imaging Platform, Université catholique de Louvain, B-1200 Brussels, Belgium; caroline.bouzin@uclouvain.be; 3 Pole of Endocrinology, Diabetes and Nutrition; Institut de Recherche Expérimentale et Clinique IREC, Université Catholique de Louvain, B-1200 Brussels, Belgium; jeanpaul.thissen@uclouvain.be; 4 WELBIO—Walloon Excellence in Life Sciences and BIOtechnology, Université catholique de Louvain, B-1200 Brussels, Belgium

**Keywords:** inulin, postprandial hypertriglyceridemia, obesity

## Abstract

Postprandial hyperlipidemia is an important risk factor for cardiovascular diseases in the context of obesity. Inulin is a non-digestible carbohydrate, known for its beneficial properties in metabolic disorders. We investigated the impact of inulin on postprandial hypertriglyceridemia and on lipid metabolism in a mouse model of diet-induced obesity. Mice received a control or a western diet for 4 weeks and were further supplemented or not with inulin for 2 weeks (0.2 g/day per mouse). We performed a lipid tolerance test, measured mRNA expression of genes involved in postprandial lipid metabolism, assessed post-heparin plasma and muscle lipoprotein lipase activity and measured lipid accumulation in the enterocytes and fecal lipid excretion. Inulin supplementation in western diet-fed mice decreases postprandial serum triglycerides concentration, decreases the mRNA expression levels of *Cd36* (fatty acid receptor involved in lipid uptake and sensing) and apolipoprotein C3 (*Apoc3*, inhibitor of lipoprotein lipase) in the jejunum and increases fecal lipid excretion. In conclusion, inulin improves postprandial hypertriglyceridemia by targeting intestinal lipid metabolism. This work confirms the interest of using inulin supplementation in the management of dyslipidemia linked to obesity and cardiometabolic risk.

## 1. Introduction

According to the World Health Organization (WHO), cardiovascular diseases (CVDs) are the first cause of death since an estimated 17.7 million people died from CVDs in 2015. As part of the metabolic syndrome (MetS), dyslipidemia linked to obesity is an important risk factor for developing CVDs [1]. A dyslipidemic profile includes hypertriglyceridemia, low HDL level and elevated level of LDL particles [1,2]. Among these factors, non-fasting triglycerides have been increasingly proposed as an important marker of the cardiovascular risk [3,4]. In fact, the human body is exposed to circulating lipids throughout the day and the postprandial period is predominant in populations eating three times a day [4]. However, studying postprandial lipemia in humans is hampered because of the lack of standardized methodology, but also because multiple factors affect the postprandial response such as genetic factors, lifestyle and physiological and pathological conditions [5,6]. Among these, diet composition plays a key role, more precisely depending on the type and amount of fat, carbohydrates, proteins, and fibers [6].

The gut microbiota is considered an important ecosystem able to control host physiology, among others by interacting with food components [7]. Dietary fibers, like inulin-type fructans, are present in many vegetables, and can also be extracted and isolated from chicory roots, to be used as food ingredients [8]. Isolated inulin-type fructans (ITF) are considered as prebiotics. Prebiotics are defined as non-digestible compounds that through fermentation by the microorganisms in the gut, modulate the composition and/or activity of the gut microbiota, thereby conferring a beneficial physiological effect on the host [9,10]. They have been extensively studied for their ability to modulate and improve lipid metabolism in the context of obesity [11,12,13]. We have previously shown that the most studied prebiotics, inulin-type fructans, decreased plasma triglycerides by reducing hepatic de novo lipogenesis [14,15]. In obese Zucker rats, ITF supplementation decreased postprandial lipemia by an unknown mechanism [16]. However, hypertriglyceridemia observed after an acute load of combined glucose and corn oil paradoxically increased in ITF-treated rats [17]. In a broader context, Catry et al. showed that ITF improved endothelial dysfunction in n-3 PUFA-depleted Apoe^−/−^ mice by a mechanism linked to the modification of the gut microbiota composition and activity [18]. In human studies, meta-analyses of randomized control trials show contradictory results regarding lipid metabolism, making it difficult to draw a conclusion [19,20,21]. In a first meta-analysis, Brighenti et al. showed that inulin-type fructans reduced serum triacylglycerols, taking into account 15 clinical trials [19]. Furthermore, Kellow et al. showed that prebiotic supplementation (mostly ITF) reduced postprandial glucose and increased reported satiety feelings in healthy adults. However, insufficient evidence was found regarding lipid levels [20]. Finally, a recent meta-analysis including 20 randomized control trials highlighted a positive effect of ITF on LDL-c level and an improvement of HDL-c level in a subgroup of type 2 diabetic patients [21]. However, this meta-analysis was recently criticized [22]. It is very likely that the conclusion of these meta-analyses are dependent on the type of prebiotic/ITF used in the clinical trials, and the population of interest. Moreover, only a few studies were performed in the postprandial state in human and in murine models.

In postprandial state, dietary lipids reach the jejunum and are first hydrolyzed into free fatty acids (FFAs) and monoacylglycerols by pancreatic lipase [23]. Thereafter, FFAs are taken up by the enterocytes upon the contribution of fatty acid receptors and triglycerides are resynthesized before being incorporated into newly formed chylomicrons. Chylomicrons are secreted in the lymph and reach the systemic circulation through the thoracic duct. Chylomicrons are then hydrolyzed by the enzyme lipoprotein lipase and FFAs are taken up by the adipose, muscle and heart tissue. Finally, chylomicrons remnants are taken up by the liver [23].

Interestingly, even if many effects of ITF occur in the lower part of the gut, where ITF are largely fermented, we have recently shown that native inulin was able to decrease sugar digestion by acting on upper intestinal disaccharidase activity [24]. This appears as a direct effect that could be independent of the gut microbiota modulation. In the context of inflammation, inulin was also shown to have direct effects in vitro, namely by acting as a ligand of toll-like receptor 4 (TLR4) [25].

In this study, we postulated that inulin-type fructans might improve postprandial hypertriglyceridemia and modulate lipid absorption. Indeed, we demonstrate the beneficial effect of ITF supplementation on hypertriglyceridemia after an acute fat load in the context of diet-induced obesity, and we propose new mechanisms highlighting the role of inulin on the expression of genes controlling lipid metabolism at the intestinal level.

## 2. Materials and Methods

### 2.1. Animals and Diet

Thirty-six male C57BL/6J mice (9 weeks old, Janvier Labs, Le Genest St Isle, France) were housed in groups of 3 per cage in a controlled environment (12-h daylight cycle) with free access to food and water. After one-week acclimatization, the mice were divided into two groups: a control group (CT) fed with a control diet (10% kcal fat, E157452-047, Ssniff, Soest, Germany) and a western diet group (WD) (45% kcal fat, E15744-347, Ssniff, Soest, Germany). The full composition of the control and western diet are given in Supplementary Table S1. After a four-week treatment, CT and WD groups were separated in two groups (CT, CT + I and WD, WD + I respectively) and supplemented or not with 0.2 g/day per mouse of native inulin (Fibruline^®^, Cosucra, Pecq, Belgium) in the drinking water for two weeks. Food intake and water consumption were recorded twice a week. For the lipid tolerance test experiment (Section 2.3), a second group of mice was used to increase the statistical power (Total *n*: CT *n* = 17, CT + I *n* = 9, WD *n* = 18, WD + I *n* = 17). For post-heparin lipoprotein lipase activity measurement (Section 2.3), another set of 36 mice (9 mice per group (CT, CT + I, WD, WD + I) was used. Moreover, for the 24 h-feces collection (Section 2.5), two sets of cages coming from two different experiments were used (Total number of cages taken into account: CT *n* = 6, CT + I *n* = 6, WD *n* = 6, WD + I *n* = 6).

### 2.2. Ethics Statement

All mouse experiments were approved by and performed in accordance with the guidelines of the local ethics committee for animal care of the Health Sector of the Université Catholique de Louvain under the specific agreement number 2014/UCL/MD/022. Housing conditions were as specified by the Belgian Law of 29 May 2013 regarding the protection of laboratory animals (agreement number LA1230314). Every effort was made to minimize animal pain, suffering, and distress.

### 2.3. Lipid Tolerance Test

After six weeks, 16 h-fasted mice were forced-fed with olive oil (10 µL/g body weight, Sigma-Aldrich, St. Louis, MO, USA). Blood samples were taken from the tail vein at baseline (before olive oil load) and every hour for 4 h. After 4 h, mice were anesthetized with isoflurane (Forene, Abbott, Queenborough, UK) before exsanguination and tissue sampling. Glycaemia was determined using a glucometer (Roche Diagnostics, Basel, Switzerland) on blood collected from the tail just before the sacrifice. Mice were killed by cervical dislocation.

In a second experiment, 16 h-fasted mice were force-fed with olive oil and the blood was taken from the tail vein after 4 h. To measure lipoprotein lipase activity, mice were injected with heparin intraperitoneally 10 min before the blood was collected (1000 U/kg body weight, Braun Medical, Melsungen, Germany). Mice were sacrificed after 4 h.

### 2.4. Real-Time Quantitative PCR

Total RNA was isolated from tissues using the TriPure isolation reagent kit (Roche Diagnostics, Penzberg, Germany). Complementary DNA was prepared by reverse transcription of 1 µg total RNA using the Kit Reverse Transcription System (Promega, Madison, WI, USA). Real-time polymerase chain reaction (PCR) was performed with a CFX96 Touch Real-Time PCR Detection System and software (Biorad Laboratories Ltd., Hercules, CA, USA) using SYBR Green (Applied Biosystems and Eurogentec, Verviers, Belgium) for detection. All samples were run in duplicate in a single 96-well reaction plate, and data were analyzed according to the 2^−∆∆*C*T^ method. The purity of the amplified product was verified by analyzing the melting curve performed at the end of amplification. The ribosomal protein L19 (RPL19) gene was chosen as a reference gene. Primer sequences are presented in Supplementary Table S2.

### 2.5. Biochemical Analysis

Plasma triglyceride and free fatty acid concentrations were measured using kits coupling enzymatic reaction and spectrophotometric detection of reaction end-products (Diasys Diagnostic and Systems, Holzheim, Germany; Randox Laboratories Ltd., Crumlin, UK; Sigma, St. Louis, MO, USA, respectively). Lipoprotein lipase activity was measured in post-heparin plasma and in the gastrocnemius muscle (50 mg tissue homogenized in 200 µL PBS) using a fluorimetric assay kit (Abcam, ab204721, Cambridge, UK). Lipid content was measured in the liver tissue after extraction with chloroform-methanol according to the Folch method [26]. Feces were collected from the cages (24 h-period). Fecal lipids were measured after extraction with chloroform-methanol. Briefly, 100 mg of dried powder-reduced feces were homogenized in 2.6 mL of chloroform: methanol (2:1). The homogenate was filtered using a Whatman filter placed at the end of a syringe and recovered in a 15 mL falcon tube. The filtrate was washed three times with phosphate buffer. The chloroform phase was evaporated under nitrogen flux, and fecal lipids were determined by weighting each tube empty and after the evaporation.

### 2.6. Histological Analysis

Two paraffin sections of jejunum of 5 µm per mice were stained with primary antibody against perilipin-3 at dilution 1:500 (catalog abs482, Millipore, Darmstadt, Germany) followed by secondary antibody donkey anti-rabbit Dylight 594 at dilution 1:1000 (SA5-10040, Thermofisher, Waltham, MA, USA). Nuclear counterstaining was performed with Hoechst 33342. Images were captured by a Pannoramic 250 Flash III (3Dhistech, Budapest, Hungary). Percentage of staining was calculated with Visiopharm software version 6.6.3.

For hepatic lipid staining, frozen liver sections were sliced at 5 µm, treated with oil red O and scanned as previously described [27]. The lipid area was determined on whole sections using the imaging software TissueIA (version 2.0.3, Leica Biosystems, Dublin, Ireland). Pixels corresponding to the oil red O staining were selected to create a color profile. Total tissue area was defined by setting the tissue intensity threshold at 210 (grey value). Results were expressed as stained area (below threshold)/tissue area (below threshold). Two representative tissue pieces were analyzed for each mouse.

### 2.7. Statistical Analysis

Results are presented as means ± SEM. Statistical analysis was performed by two-way analysis of variance (main effect diet, treatment and diet × treatment) followed by Tukey post hoc multiple comparison tests using R software (version 1.0.136). For body weight evolution, triglyceride and free fatty acid profile, a linear mixed model was used followed by ANOVA with the use of time, diet and treatment as fixed effects and mice as a random effect. For the triglyceride profile, the factor “experiment” was added as a random effect. Tukey post hoc tests were performed at each time. The results were considered statistically significant at *p* < 0.05.

## 3. Results

### 3.1. Inulin Does Not Influence Body Weight, Adipose Tissue and Liver Weight

**Figure 1:**
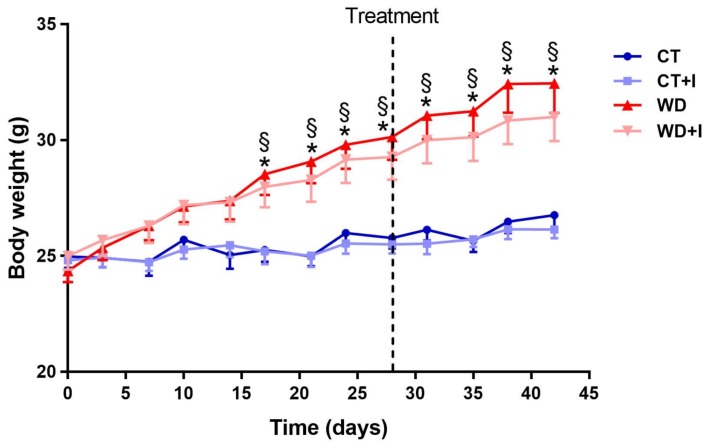
Body weight evolution. Inulin supplementation was introduced after 4 weeks. Data are presented as mean ± SEM and analyzed with a mixed model ANOVA followed by Tukey post hoc test. * *p* < 0.05 versus CT, § *p* < 0.05 versus CT + I.

Mice were fed a WD or CT diet for six weeks and supplemented or not with inulin for the last two weeks of the experiment. After 17 days and until the end of the experiment, western diet induced a significant increase in body weight. Supplementation with inulin for the last two weeks of the experiment was not able to counteract the increase in body weight observed after six weeks of western diet (Figure 1). Similarly, adipose tissues weights were increased by the western diet. Inulin supplementation did not impact tissues weights (Table 1). The cecal tissue weight was increased with inulin supplementation (in CT + I and WD + I compared to CT and WD groups), whereas the cecal content was increased in WD + I versus WD and CT groups (Table 1).

### 3.2. Inulin Supplementation Improves Postprandial Hypertriglyceridemia Induced by Western Diet

**Figure 2:**
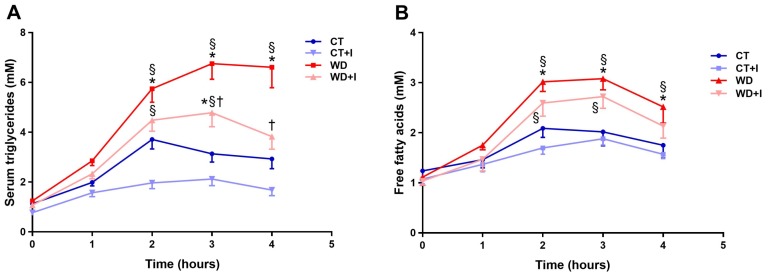
Serum triglyceride profile after olive oil load (**A**). Free fatty acid profile after olive oil load (**B**). Data are presented as mean ± SEM and analyzed with a mixed model ANOVA followed by Tukey post hoc tests. * *p* < 0.05 versus CT, § *p* < 0.05 versus CT + I, † *p* < 0.05 versus WD.

In order to assess the effect of inulin on postprandial lipid metabolism, we performed a lipid tolerance test and we evaluated serum triglycerides and free fatty acids concentration over a 4-h period. Compared to CT, WD induced a significant increase in serum triglycerides after olive oil load (Figure 2A). Inulin supplementation in western diet led to a significant improvement of hypertriglyceridemia after three and four hours post-lipid load. Free fatty acid concentration displayed similar trends as the triglycerides measurement (Figure 2B). However, inulin did not significantly influence free fatty acid concentration. Moreover, fasting glycemia was impacted neither by the diet nor by the treatment (Supplemental Figure S1).

### 3.3. Inulin Modifies Intestinal Lipid Metabolism by Changing Gene Expression in the Jejunum

#### 3.3.1. Inulin Decreases *Cd36* Expression, a Receptor Involved in Fatty Acid Absorption and Lipid Sensing

**Figure 3:**
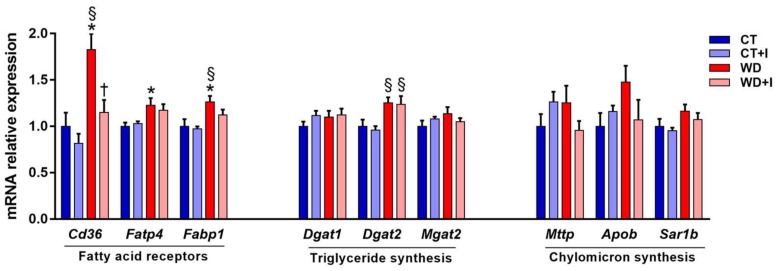
mRNA relative expression of genes involved in fatty acid absorption in the jejunum. Data are presented as mean ± SEM and analyzed with two-way ANOVA followed by Tukey post hoc tests. * *p* < 0.05 versus CT, § *p* < 0.05 versus CT + I, † *p* < 0.05 versus WD.

Among the mechanisms responsible for hypertriglyceridemia, lipid absorption and sensing at the intestinal level can be pointed out as important targets. After 6 weeks of western diet, the expression of several markers involved in fatty acid absorption and lipid sensing (*Cd36*, *Fatp4*, *Fabp1*), triglyceride synthesis (*Dgat2*, main effect diet *p*-value < 0.05) and chylomicron synthesis (*Sar1b*, main effect diet *p*-value < 0.05) were increased in WD compared to CT (Figure 3). Interestingly, inulin supplementation was able to restore *Cd36* mRNA expression at a level comparable to control in the WD + I group.

#### 3.3.2. Inulin Supplementation Increases Fecal Lipid Excretion When Fed with Western Diet

**Figure 4:**
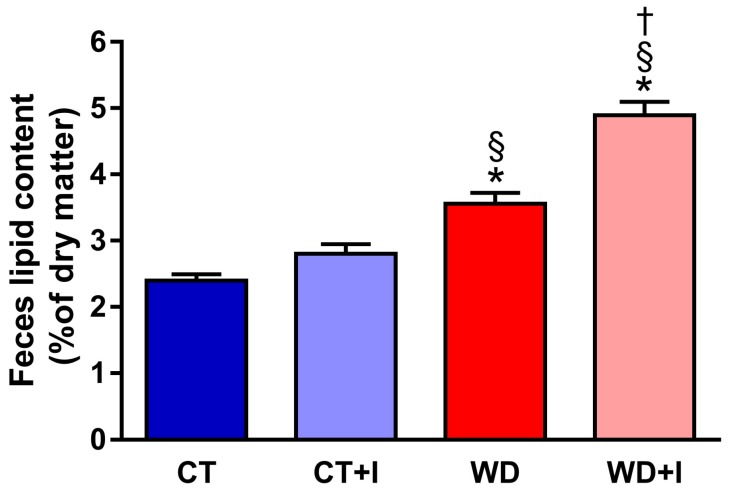
Fecal lipid content. Data are presented as mean ± SEM and analyzed with two-way ANOVA followed by Tukey post hoc tests. * *p* < 0.05 versus CT, § *p* < 0.05 versus CT + I, † *p* < 0.05 versus WD.

To assess if inulin supplementation lessened lipid absorption, fecal lipid excretion was measured in the different groups (Figure 4). Compared to CT, WD feces had a higher content in total lipids and inulin supplementation further increased fecal lipid content in WD + I groups (main effects *p*-value diet < 0.001, treatment < 0.001, diet × treatment < 0.001).

#### 3.3.3. Western Diet and Inulin Supplementation Does Not Impact Lipid Accumulation in the Enterocytes

Fatty acid accumulation in the enterocytes can affect postprandial lipid excursion in the blood. Therefore, we measured perilipin-3 staining in the jejunum of mice 4 h following the lipid tolerance test (Supplemental Figure S2A,B). Compared to controls, no effect on fatty acid accumulation was observed when mice were fed a western diet. Furthermore, inulin supplementation did not impact fatty acid accumulation either in the CT diet or in the WD-fed mice.

### 3.4. Effect of Dietary Intervention on Lipoprotein Lipase Activity and on the Expression of Genes Involved in Its Regulation

**Figure 5:**
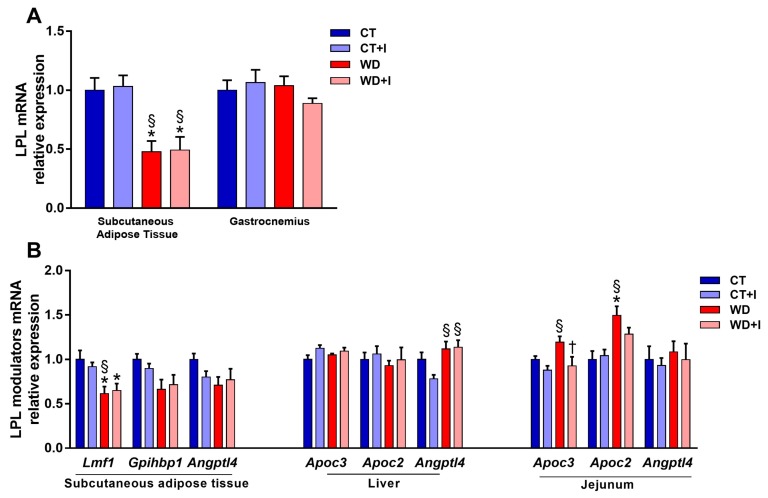
Lpl mRNA expression in the muscle and subcutaneous adipose tissue (**A**). Level of mRNA expression of post-translational regulators of LPL activity (**B**). Data are presented as mean ± SEM and analyzed with two-way ANOVA followed by Tukey post hoc tests. * *p* < 0.05 versus CT, § *p* < 0.05 versus CT + I, † *p* < 0.05 versus WD.

Lipoprotein lipase is an important enzyme in the postprandial metabolism of fat that is responsible for the hydrolysis of triglyceride-rich lipoproteins. The downregulation of its expression or activity can cause dyslipidemia [1]. Therefore, we measured *Lpl* mRNA expression in different tissues. Compared to CT, WD-fed groups had a lower level of expression of *Lpl* in the subcutaneous adipose tissue whereas no effect of the diet was seen in the muscle (Figure 5A). Inulin supplementation did not change the level of *Lpl* mRNA expression either in the adipose tissue or in the muscle (Figure 5A). We measured the expression of several genes responsible for the post-translational regulation of LPL activity in the jejunum, the liver and the subcutaneous adipose tissue (Figure 5B). Compared to CT, the expression of Lipase maturation factor 1 (*Lmf1*) is reduced in the subcutaneous adipose tissue of WD and WD + I, in accordance with the reduction of *Lpl* mRNA expression. Moreover, *Gpihbp1* (GPI-anchored protein, required for the transport and stabilization of LPL to the surface of endothelial cells [2]) is also reduced in WD and WD + I compared to CT (main effect *p*-value diet < 0.05). In the liver, fasting-induced adipose factor (*Fiaf*/*Angptl4*) is increased both in WD + I and WD compared to CT + I, an effect that could contribute to the inhibition of LPL activity. However, in the jejunum, the level of *Apoc3* mRNA expression (inhibitor of LPL) was significantly decreased in inulin-treated groups (main effect *p*-value treatment < 0.05) whereas the level of *Apoc2* (activator of LPL) was increased in WD groups (main effect *p*-value diet < 0.05). Post-heparin total LPL activity was measured 4 h after the lipid load (Supplemental Figure S3A). Total circulating LPL activity was not impacted by the diet or the treatment. Moreover, lipoprotein lipase activity was measured in the muscle after 4 h of lipid load (Supplemental Figure S3B). LPL activity was decreased in WD-fed mice compared to CT-fed mice (diet main effect *p*-value < 0.05), whereas inulin supplementation did not affect its activity.

### 3.5. Inulin Decreases Hepatic Lipid Content and Changes the Hepatic Expression of Genes Involved in VLDL Secretion

**Figure 6:**
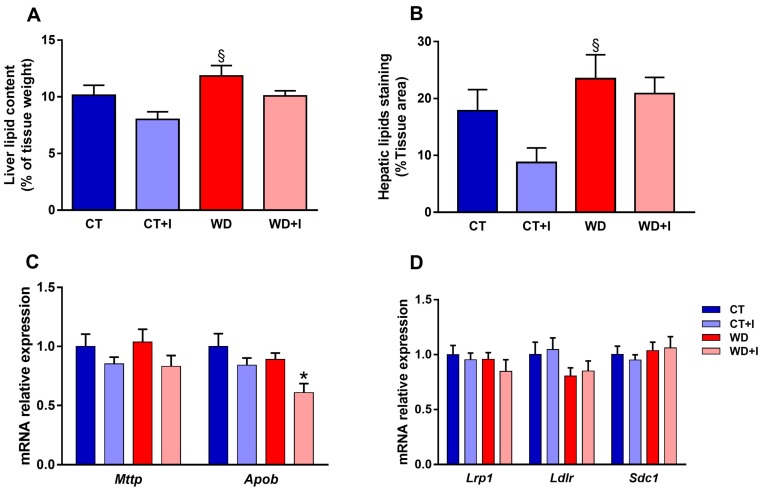
Liver lipid content (**A**). Quantification of hepatic lipid staining with oil red O (**B**). Level of mRNA expression of *Mttp* and *Apob*, involved in VLDL synthesis in the liver (**C**). Level of mRNA expression of remnant receptors *Lrp1*, *Ldlr* and *Sdc1* in the liver (**D**). Data are presented as mean ± SEM and analyzed with two-way ANOVA followed by Tukey post hoc tests. * *p* < 0.05 versus CT, § *p* < 0.05 versus CT + I.

The obese phenotype is often associated with lipid accumulation in the liver. We measured liver lipid content (Figure 6A) and percentage staining (Figure 6B, illustrated in Supplemental Figure S4) in the four groups. Compared to control, there was a small accumulation of lipids in the liver of western diet-fed mice (main effect diet *p*-value < 0.05) (Figure 6A). Inulin supplementation slightly decreased lipid accumulation in CT + I and WD + I fed mice (main effect treatment *p*-value < 0.05) (Figure 6A). The measure of the percentage of lipid staining followed the same trend (main effect diet *p*-value < 0.05, main effect treatment *p*-value = 0.1). VLDL secretion by the liver participates to some extent to postprandial lipemia. In this regard, we measured mRNA expression level of apolipoprotein B (*Apob*) and microsomal triglycerides transfer protein (*Mttp*) in the liver (Figure 6C). Inulin treatment significantly decreased *Apob* mRNA expression (treatment main effect *p* < 0.05) and tended to decrease *Mttp* (treatment main effect *p*-value = 0.06). Remnant particles are cleared from the blood by the liver with the help of specific receptors (LDL receptor, Syndican 1, LDL receptor-related protein 1). We measured the level of mRNA expression of those receptors in the liver but neither the diet nor the treatment with inulin had any effect on their relative expression (Figure 6D).

## 4. Discussion

Dyslipidemia induced by western diet is a key component of the metabolic syndrome, in addition to insulin resistance, type 2 diabetes, hypertension, obstructive sleep apnea syndrome and non-alcoholic fatty liver disease [1]. Altogether, these factors are predictive of cardiovascular disease risks [1]. In this study, we demonstrated that inulin supplementation can reverse the hypertriglyceridemia driven by an acute lipid challenge in mice chronically fed a western diet. This effect is independent of weight loss, since body weight, adipose tissue and liver weights were not modified throughout the intervention. Longer treatment might have had an effect on adipose tissues weight, as shown in other studies using oligofructose (OFS) supplementation [28,29]. These results confirm the beneficial effect of ITF supplementation in the context of altered lipid metabolism [12,16,18]. We also highlighted the potential mechanisms implicated in the improvement of diet-induced lipid homeostasis disturbances by inulin. After inulin supplementation, we showed that *Cd36* mRNA expression in the jejunum was restored at levels comparable to CT. CD36 plays a key role in the enterocytes as a transporter for long chain fatty acids (LCFA) and as a lipid sensor allowing chylomicron formation [30,31]. In fact, Tran et al. showed that in the presence of LCFA, CD36 is degraded and leads to the decrease of ERK1/2 activation, which further increases MTTP protein, required for apolipoprotein B48 lipidation [31]. Thus, they show that 4 h after an acute lipid challenge, *Cd36* mRNA expression is significantly decreased compared to baseline in lean mouse [31]. Furthermore, in a mouse model of metabolic syndrome (MetS), it was shown that MetS mice displayed accentuated postprandial hypertriglyceridemia after 3 h due to a defective clearance of triglyceride-rich lipoproteins and that this was associated in a delay in the induction of MTTP, LFABP, and APOC2 linked to blunted lipid sensing by CD36 in that model. In MetS mice, CD36 was not downregulated by lipids, in contrast to control mice [32]. In line with these results, we hypothesized that CD36 lipid sensing was restored by inulin in WD-fed mice as the expression of *Cd36* was decreased in WD + I compared to WD after 4 h of lipid challenge. However, we could not relate these data with any effect of inulin supplementation on the mRNA expression of key genes involved in chylomicrons formation and clearance such as *Mttp*, *Lfabp* or *Apoc2*, as shown by Buttet et al. after 1 h of a lipid load [32].

Also, it was shown that the oral administration of small inhibitors of the LCFA-CD36 binding significantly reduces the postprandial hypertriglyceridemia which follows a gastric olive oil challenge [33]. The decrease of *Cd36* mRNA expression by inulin supplementation might, therefore, be related to the improvement of serum triglycerides after an acute lipid challenge.

In the second part of this work, we show that inulin supplementation increases fecal total lipid content, independently of the acute lipid challenge. Several mechanisms have been proposed to explain the potential of dietary fibers to decrease lipid digestibility. In fact, some dietary fibers, due to their viscous state, can drastically reduce the rate of lipid emulsification, with a resulting noticeable lowering of the extent of fat lipolysis in the intestine [34]. Moreover, in a recent study, Suriano et al. showed that other types of fibers, namely wheat bran, also increased fecal lipids content in the context of diet-induced obesity, due to an increased in the fat binding capacity of wheat bran [27]. However, since ITF are non-viscous fibers and have a weak fat binding capacity [35], we propose that this effect could be related to the decrease in the mRNA expression of the fatty acid receptor *Cd36*. However, it was shown that genetic deletion of *Cd36* does not affect intestinal lipid uptake [36,37]. Rather, CD36 is recognized as an important lipid sensor for chylomicrons formation and secretion [36]. Therefore, the link between the drop in *Cd36* mRNA expression and the increased fecal lipid excretion should be further investigated.

The accumulation of dietary lipids in the enterocytes can also play a role in the extent of hypertriglyceridemia observed after an acute lipid challenge [38]. However, in this study, we did not observe any significant effect of WD or inulin supplementation on lipid accumulation in the enterocytes.

It is well recognized that lipolysis of triglyceride-rich lipoproteins is impaired in obesity and co-occurs with reduced mRNA expression of *Lpl* in adipose tissue and reduction of LPL activity in skeletal muscle [1]. In this study, we obtain similar results by showing that WD decreases mRNA expression of *Lpl* in adipose tissue and activity in skeletal muscle. In accordance with *Lpl* mRNA level in adipose tissue, we observed a decrease of two post-translational regulators of LPL activity, namely *Lmf1* and to a lesser extent *Gpihbp1*. However, we could not relate this to a change in total post-heparin LPL activity. Contradicting data exist on the impact of inulin supplementation on *Lpl* mRNA expression in different tissues. Prebiotic supplementation was shown to increase muscle *Lpl* mRNA expression in mice fed an HFD supplemented with oligofructose [29], whereas in another study, *Lpl* mRNA expression was significantly decreased by oligofructose supplementation in the subcutaneous adipose tissue [28]. In these studies, the supplementation with oligofructose was associated with a significant decrease in fat mass gain [28,29]. In our study, inulin supplementation did not impact *Lpl* mRNA expression in the adipose tissue or the muscle. However, at the intestinal level, we observe a small but significant reduction of *Apoc3* levels. Apolipoprotein C3 acts as an inhibitor of LPL activity by disabling the attachment of triglyceride-rich lipoproteins to the cell surface [39]. The link between APOC3, hypertriglyceridemia and cardiovascular risk has been confirmed in extensive animal and humans studies, and abundant data support that the reduction in circulating levels of APOC3 levels (e.g., using anti-sense oligonucleotide targeting the hepatic mRNA of *Apoc3* [40]) may represent a valid target for hypotriglyceridemic therapy [39,41]. In our case, we were unable to relate the decrease of *Apoc3* mRNA expression with an improvement of triglyceride-rich lipoprotein lipolysis. Nonetheless, this further reinforces our hypothesis that the mechanisms participating to the improvement of postprandial lipemia by ITF supplementation are most likely partly located in the gut and more precisely in the jejunum, where lipid absorption occurs.

Hepatic remnant clearance is a process implicating three different receptors: LRP1, LDLR, SDC1. Deletion or deregulation of these receptors leads to abnormal remnant clearance [42]. However, those factors were not modulated at the level of mRNA expression by the dietary intervention, suggesting that those processes are not involved in the improvement of hypertriglyceridemia. A direct measurement of hepatic remnant clearance would be needed to confirm this hypothesis.

On the other hand, at the hepatic level, we evaluated whether western diet and inulin supplementation impacted lipid accumulation and the secretion of VLDL. In fact, although about 80% of the increase of triglycerides after a fat-loaded meal comes from chylomicrons, approximately 80% of the increase in particle number is accounted for by VLDL particles coming from the liver [3]. We found comparable mRNA levels of *Mttp* and *Apob* between control and western diet groups and inulin supplementation slightly decreases *Apob* levels. Thus, a small decrease of VLDL secretion could participate to the hypotriglyceridemic effect of inulin supplementation. These results are in line with a previous study showing that oligofructose reduces serum VLDL concentration in rats [43], due to a decrease enzymatic activity of the fatty acid synthase, a key enzyme involved in lipogenesis [15]. To further prove that a decreased VLDL secretion is directly implicated in decreased postprandial lipemia, isolated hepatocytes from mice previously force-fed with oil could be used as previously described in rats with OFS supplementation [15,43]. Moreover, we found a small decrease of hepatic lipid accumulation in inulin-treated mice, as previously demonstrated with oligofructose supplementation [14,15,17,44].

It is not excluded that inulin can directly target the upper intestinal cells. Indeed, inulin can have direct effects on specific targets such as for example on the inhibition of disaccharides activity [24] but also the modulation of inflammation by acting as a ligand for TLR4 [25]. Thus, inulin could exert metabolic regulation independently of the modulation of the colonic microbiota by interacting directly with intestinal epithelial cells. Moreover, inulin could exert its beneficial effect through the modulation of the small intestine microbiota, also involved in the regulation of key metabolic function [45]. However, no study until now has ever evaluated the impact of inulin supplementation on small intestinal microbiota composition and activity.

Another possible mechanism involved in the improvement of hypertriglyceridemia by ITF could implicate bile acid metabolism and signaling. ITF have been shown to affect bile acid profile in the gallbladder, caecum and in the systemic and portal circulation [18,46,47]. Bile acids are amphipathic molecules important for the solubilization of dietary fat, but can also act as signaling molecules of specific receptors, namely farsenoid X receptor and the G-coupled receptor protein TGR5 [48]. Increasing evidence shows that bile acids and their receptors could play a key role in the modulation of dyslipidemia [49]. However, further studies are needed to unravel the pathways linking the improvement of hypertriglyceridemia with the modulation of the bile acid profile by ITF.

## 5. Conclusions

In conclusion, we show that inulin supplementation decreases hypertriglyceridemia induced by a western diet after an acute lipid load. Mechanisms implicated involve a potential improvement of lipid sensing in the jejunum and an increase in fecal lipid excretion. These data confirm the interest of using ITF supplementation in the management of dyslipidemia linked to obesity and cardiovascular diseases.

## Figures and Tables

**Table 1:** Organ weights.

Tissues	CT	CT + I	WD	WD + I
Liver (g)	0.87 ± 0.04	0.89 ± 0.02	1.00 ± 0.05	0.91 ± 0.05
Subcutaneous adipose tissue (g)	0.41 ± 0.05	0.29 ± 0.01	0.85 ± 0.13 *^,§^	0.75 ± 0.12 ^§^
Epididymal adipose tissue (g)	0.40 ± 0.04	0.28 ± 0.01	1.00 ± 0.17 *^,§^	0.88 ± 0.17 *^,§^
Visceral adipose tissue (g)	0.18 ± 0.02	0.12 ± 0.01	0.37 ± 0.06 *^,§^	0.29 ± 0.05 ^§^
Cecal tissue (g)	0.039 ± 0.002	0.054 ± 0.002 *	0.033 ± 0.002 ^§^	0.046 ± 0.003 ^†^
Cecal content (g)	0.15 ± 0.01	0.19 ± 0.01	0.18 ± 0.01	0.23 ± 0.02 *^,†^

Data are presented as mean ± SEM and analyzed with a two-way ANOVA followed by Tukey post hoc tests. * *p* < 0.05 versus CT, ^§^ *p* < 0.05 versus CT + I, ^†^ *p* < 0.05 versus WD.

## Supplementary Materials

The followings are available online at http://www.mdpi.com/2072-6643/10/5/532/s1, Figure S1: Fasting glycemia Figure S2: Perilipin-3 staining in jejunum (A) Quantification of percentage of staining area (B), Figure S3: Post-heparin lipoprotein lipase activity (A) Lipoprotein lipase activity in the gastrocnemius muscle (B), Figure S4: Hepatic lipid staining with oil red O. Table S1: Composition of the diets, Table S2: Sequences of primers used for real-time quantitative PCR.
