# Supplementary material for: Inulin Improves Postprandial Hypertriglyceridemia by Modulating Gene Expression in the Small Intestine
Source: Nutrients. 2018 Apr 25;10(5):532. doi: 10.3390/nu10050532 (PMC5986412; doi:10.3390/nu10050532)
Supplement: Supplementary file 1 [file nutrients-10-00532-s001.zip › Supplementary_file/Supplementary Material.docx]

Supplementary Material

**Figure S1**. Fasting glycemia. Data are presented as mean ± SEM.

**
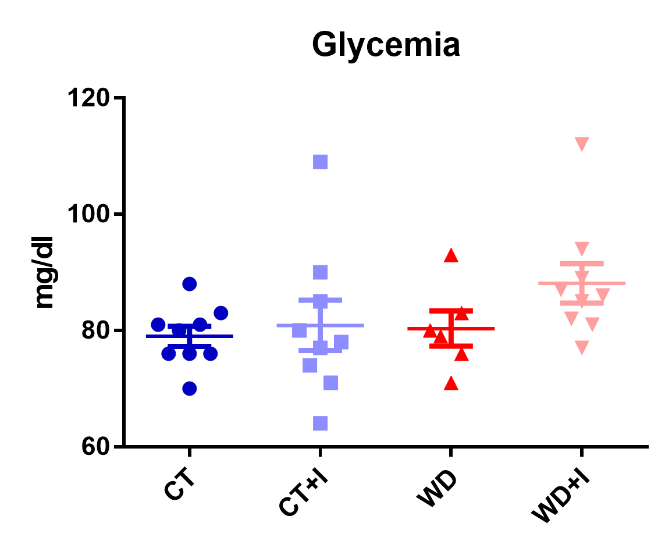
**

**Figure S2.** Perilipin-3 staining in jejunum (A) Quantification of percentage of staining area (B). Data are presented as mean ± SEM. Scale bar=100 µm.

**A**


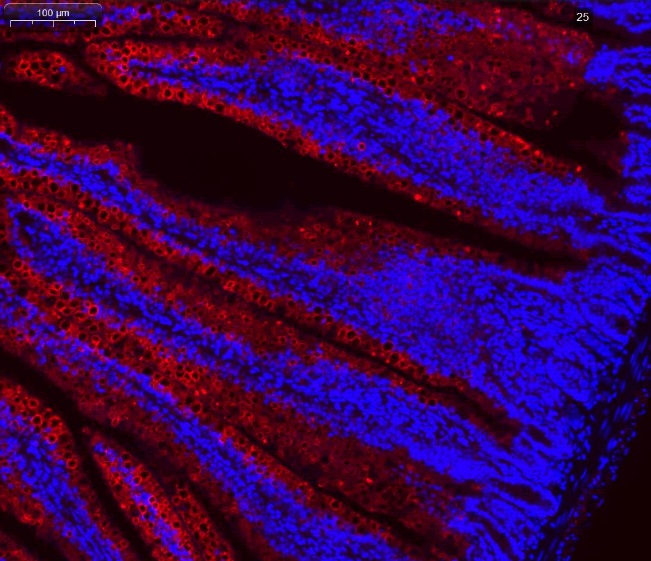

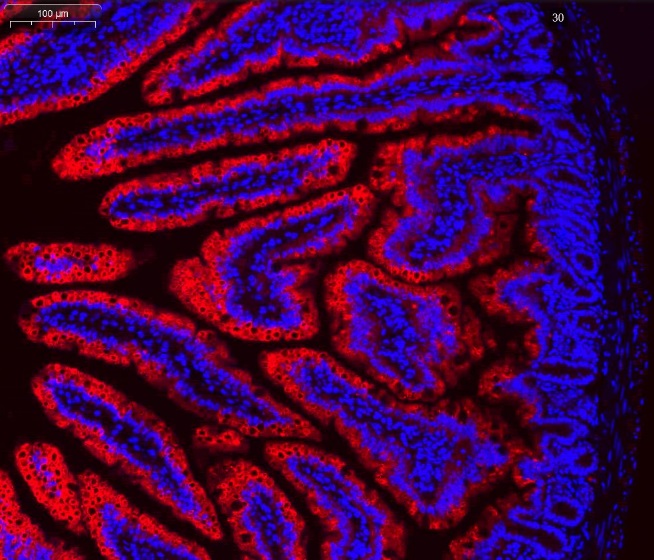

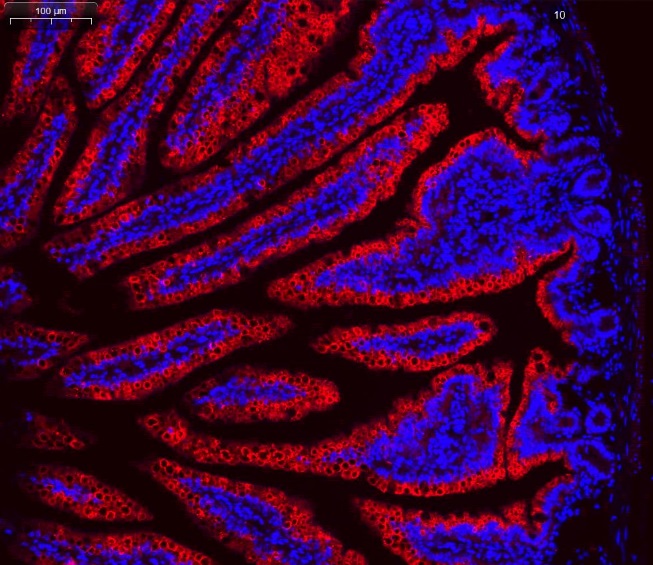

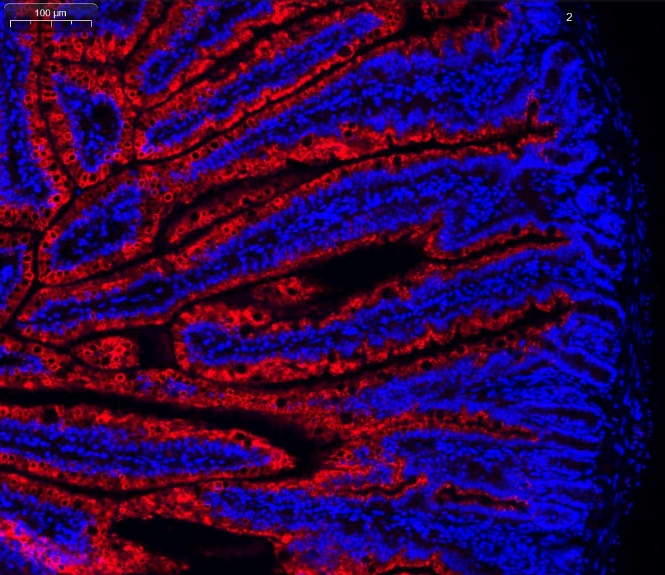


CT

WD

WD+I

WD

CT+I

**B**


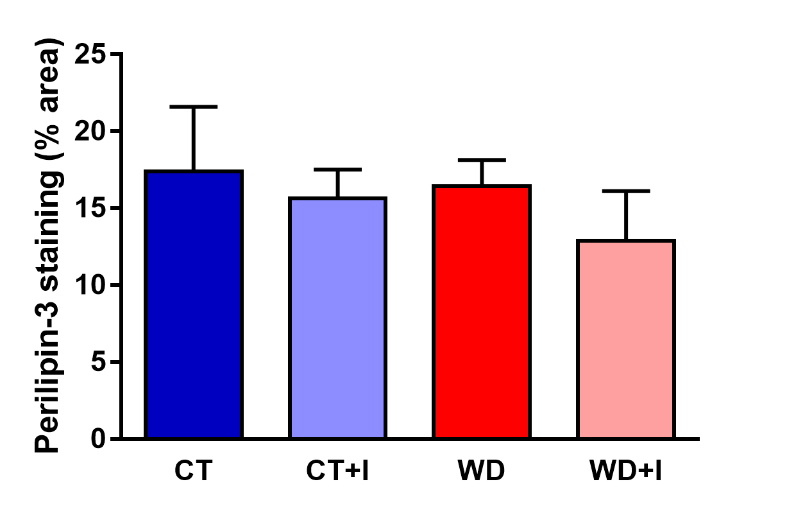


**Figure S3.** Post-heparin lipoprotein lipase activity (A) Lipoprotein lipase activity in the gastrocnemius muscle (B). Data are presented as mean ± SEM.

**
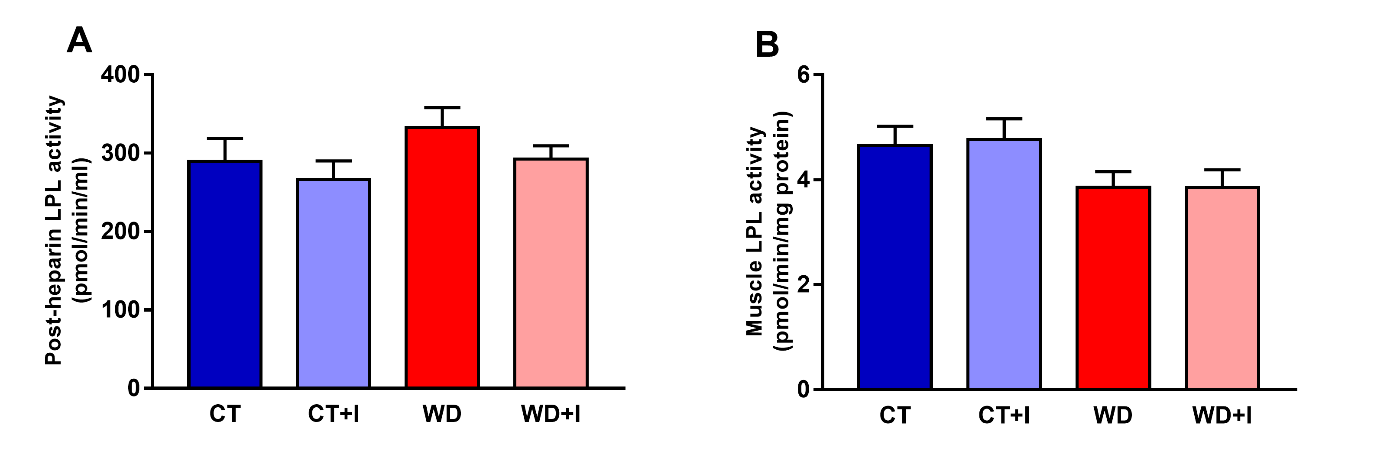
**

**Figure S4.** Hepatic lipid staining with oil red O. Scale bar=100µm

**
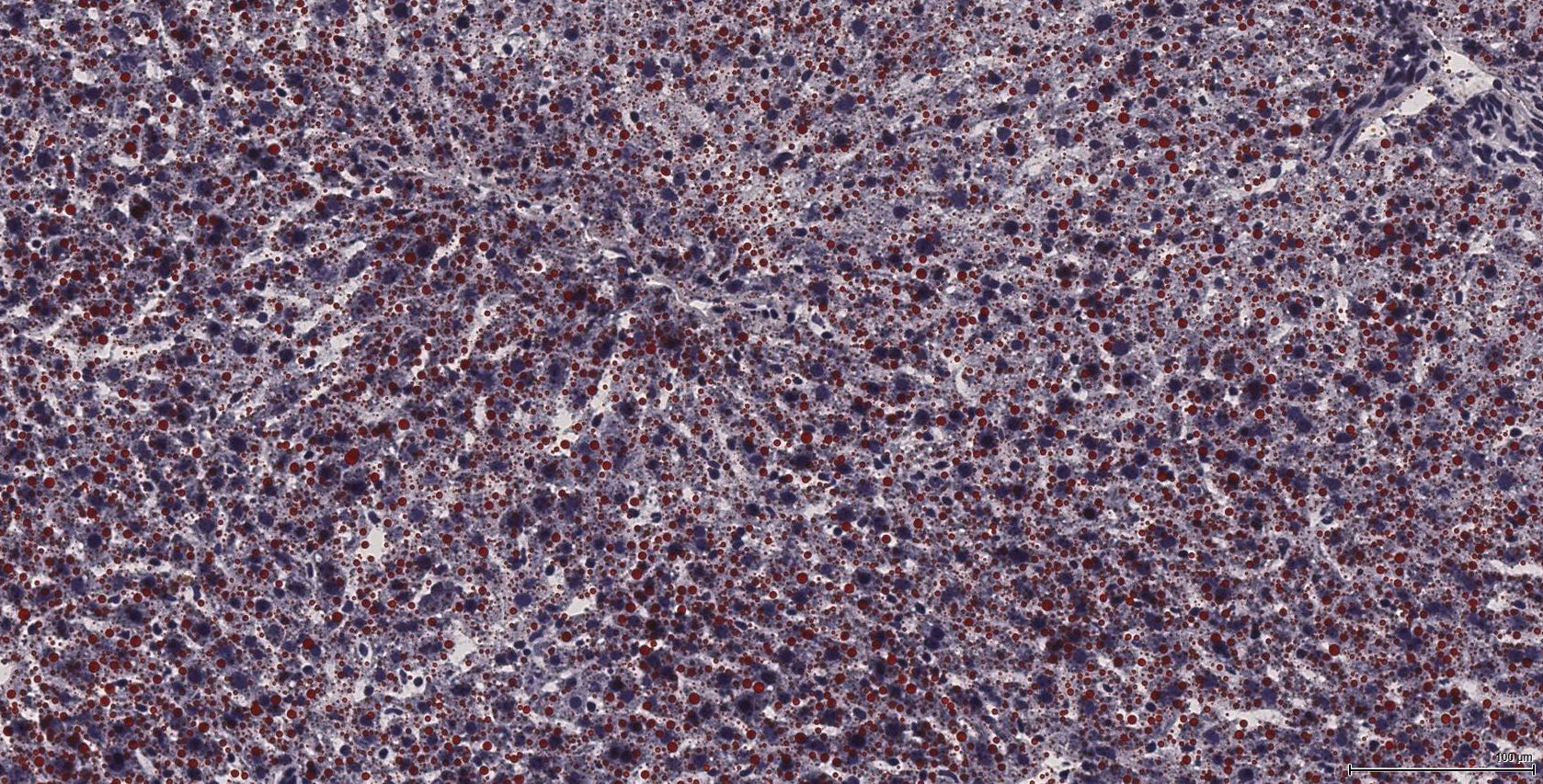
**
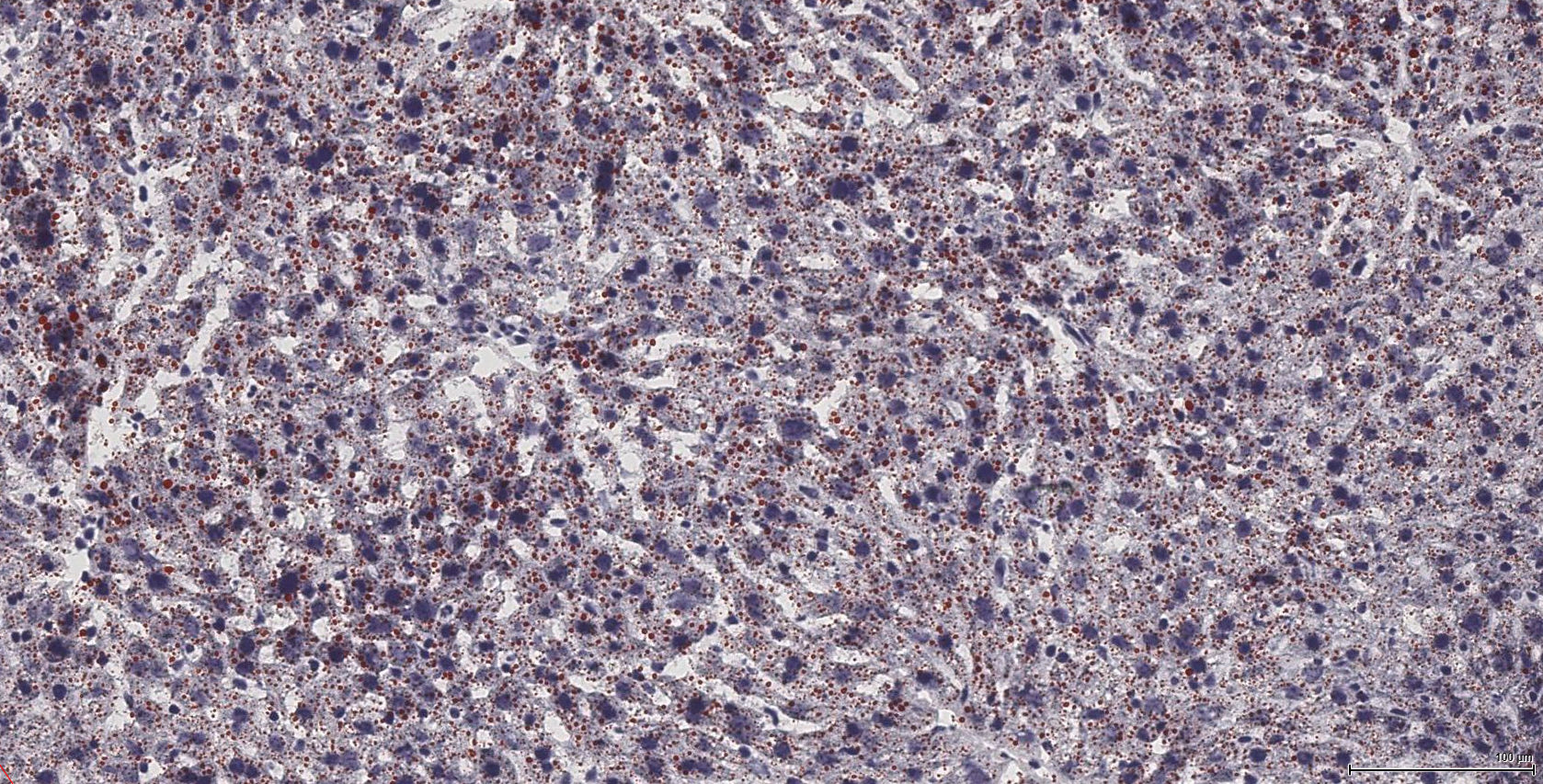


CT+I

CT

**
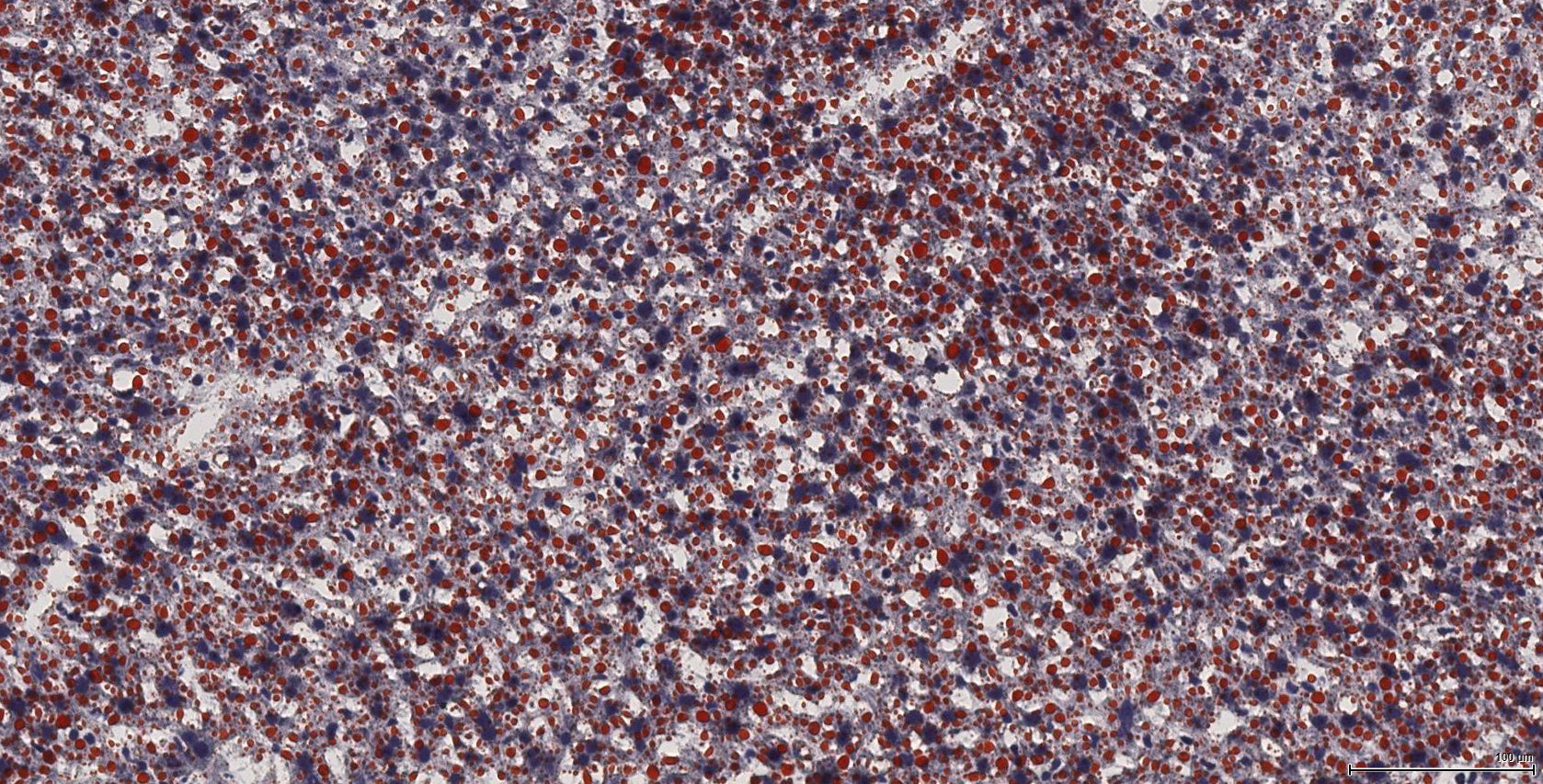

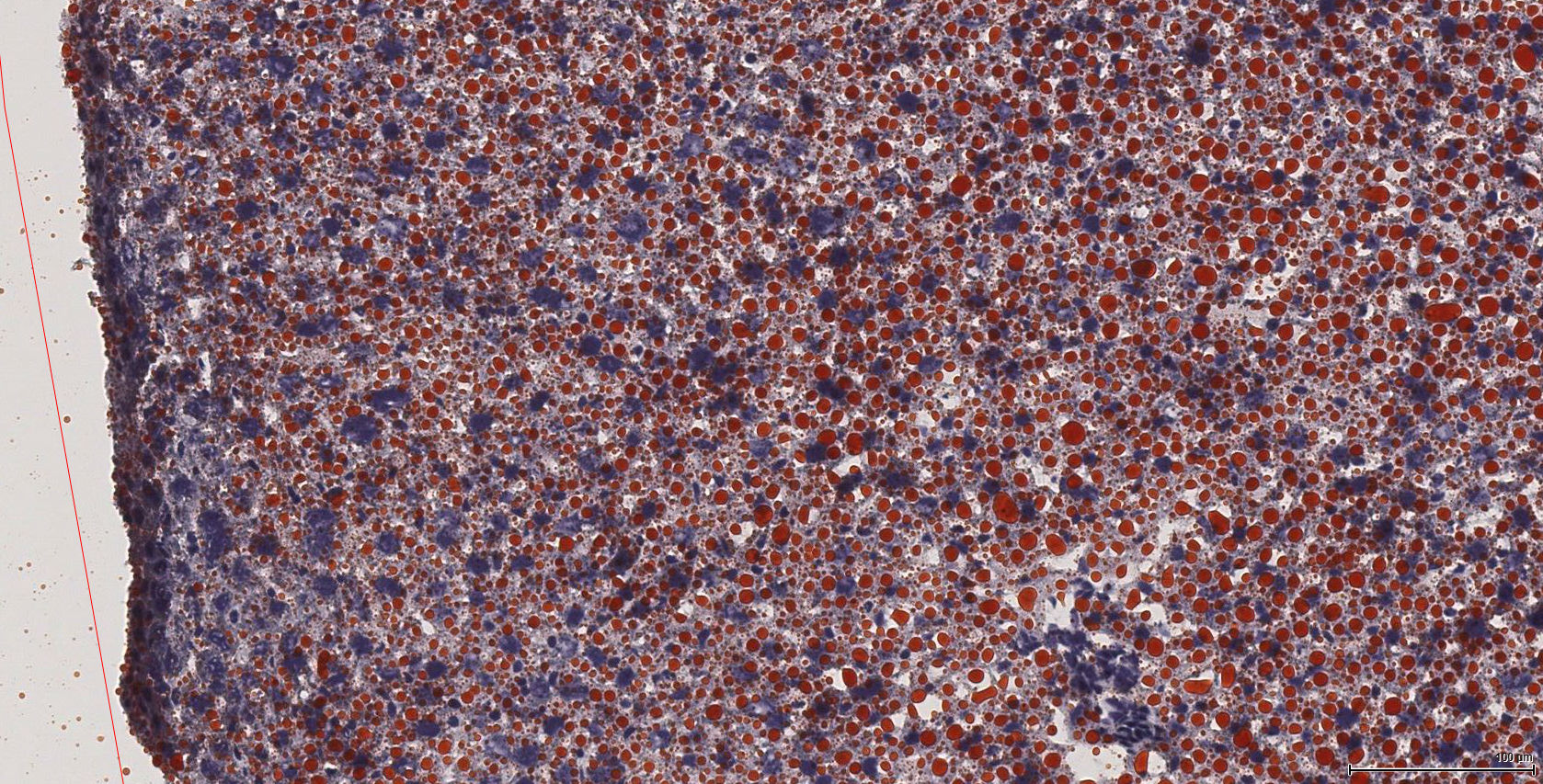
**

WD+I

WD
