# Supplementary material for: Inulin Improves Postprandial Hypertriglyceridemia by Modulating Gene Expression in the Small Intestine
Source: Nutrients. 2018 Apr 25;10(5):532. doi: 10.3390/nu10050532 (PMC5986412; doi:10.3390/nu10050532)
Supplement: Supplementary file 1 [file nutrients-10-00532-s001.zip › Supplementary_file/Table S1. Composition of the diets.docx]

**Table S1. Composition of the diets**

| **Feed composition (%)** | WD (E15744-347) | CT (E157452-047) |
| --- | --- | --- |
| Casein | 25 | 20.7 |
| Pork lard | 20.8 | 1.6 |
| Soybean oil | 2.8 | 2.4 |
| Corn starch | 7 | 48.85 |
| Maltodextrin | 11 | 14 |
| Sucrose | 20.17 | - |
| Cellulose powder | 5.7 | 5 |
| L-Cystine | 0.3 | 0.25 |
| Vitamine premix | 1 | 1 |
| Mineral & trace element premix | 6 | 6 |
| Choline chloride | 0.2 | 0.2 |
| Dye (red) | 0.03 | - |

| **Energy content (% kJ)** | WD (E15744-347) | CT (E157452-047) |
| --- | --- | --- |
| Fat | 45 | 10 |
| Protein | 20 | 20 |
| Carbohydrates | 35 | 70 |
