# Supplementary material for: Inulin Improves Postprandial Hypertriglyceridemia by Modulating Gene Expression in the Small Intestine
Source: Nutrients. 2018 Apr 25;10(5):532. doi: 10.3390/nu10050532 (PMC5986412; doi:10.3390/nu10050532)
Supplement: Supplementary file 1 [file nutrients-10-00532-s001.zip › Supplementary_file/Table S2. Sequences of primers used for real-time quantitative PCR.doc]

**Table S2. Sequences of primers used for real-time quantitative PCR**

|  | **Forward primer** | **Reverse primer** |
| --- | --- | --- |
| ***APOA4*** | ATGCCAAGGAGGCTGTAGAA | CTGTACGACAAAGGGCACCA |
| ***APOB*** | GGAAGCTGTTGCTGAGTGGT | ACCCTTCTCTGATGGCCTGT |
| ***APOC2*** | AGAAGACATACCCGATCAGCA | TCCCCTCAGGAGAGTAAGGAG |
| ***APOC3*** | CGCTAAGTAGCGTGCAGGAG | GGTTGGTCCTCAGGGTTAGA |
| ***CD36*** | GCCAAGCTATTGCGACATGA | ATCTCAATGTCCGAGACTTTTCAAC |
| ***DGAT1*** | CGTGGTATCCTGAATTGGTGT | AGGGGTCCTTCAGAAACAGAG |
| ***DGAT2*** | ACTCTGGAGGTTGGCACCAT | GGGTGTGGCTCAGGAGGAT |
| ***FABP1*** | GCAGAGCCAGGAGAACTTTG | TGATGTCCTTCCCTTTCTGG |
| ***FATP4 (SLC27A4)*** | AACAGCGGGTCTTTCACAAC | TTGCAAGTCCCATCAGCAAC |
| ***ANGPTL4*** | CAATGCCAAATTGCTCCAATT | TGGCCGTGGGCTCAGT |
| ***GPIHBP1*** | AGGAAGAGGAGGAGACCAACA | GACGAGCGTGATGCAGAAG |
| ***LDLR*** | CTGTGGGCTCCATAGGCTATCT | GCGGTCCAGGGTCATCTTC |
| ***LMF1*** | CCTGGCCCTACTTGCTGTT | CTTGCGGATCCACCACTT |
| ***LRP1*** | ACACGGGCACAATCTCCTAC | GATACCCCTCGGCATCTTC |
| ***LPL*** | TCTGTACGGCACAGTGG | CCTCTCGATGACGAAGC |
| ***MGAT2 (MOGAT2)*** | GAATCGCAAGGGCTTCATC | CAGGTACCAGGGGTGTTCTC |
| ***MTTP*** | ATGATCCTCTTGGCAGTGCTT | TGAGAGGCCAGTTGTGTGAC |
| ***SAR1B*** | GAAGCCATCAGTGAAGAGAGGT | CCATAGCCCTGCCTTTTTAG |
| ***SDC1*** | TGGCTGTAAATGTTCCTCCT | GTCAACAGCCACACGTCCTT |
